# Message from animal models and experimental medicine for 2024—Striving for excellence with distinctive features

**DOI:** 10.1002/ame2.12390

**Published:** 2024-03-25

**Authors:** Chuan Qin

**Affiliations:** ^1^ Chinese Association for Laboratory Animal Sciences Beijing China; ^2^ Institute of Laboratory Animal Sciences Chinese Academy of Medical Sciences & Peking Union Medical College Beijing China

Since its inception, *Animal Models and Experimental Medicine (AMEM)* has received 632 articles in total from 52 countries and regions including China, Iran, the United States, India, Nigeria, Israel, Germany, Iraq, Italy, Japan, Australia, Bangladesh, Belgium, Brazil, and Canada, among others. AMEM has become an important international exchange platform for innovative research achievements in the field of laboratory animal science and basic medicine. In 2023, we were pleased to see that the total number of published articles in AMEM reached 274, and the number of publications from international groups increased. And we hope that this proportion will continue to rise in the future.

In 2023, based on maintaining high citation rates in the themed sections on neurodegenerative diseases and cardiovascular and cerebrovascular diseases, AMEM added three new international hot topics: multi‐omics data analysis of animal models, usage of different tumor models in cancer research and the role of regulatory non‐coding RNA in human diseases. In April 2023, a successful AMEM editorial board meeting was held in Taiyuan. During this meeting, the executive editor, associate editor, all the editorial board members and the editorial department actively exchanged views and contributed to the development of AMEM. At the meeting, I presented various awards to the editorial board members who had supported AMEM development from the very beginning, including the 2018–2020 AMEM Excellent Paper award, the Excellent Editorial Board Member award, the Excellent Reviewer award, the Publicity Ambassador award and the Outstanding Contribution Award for Editorial Board Members. AMEM appreciates the dedicated efforts of all editorial board members and hopes that we can work together in the new year to attain a brighter future.

For AMEM, the key to standing out from the rest of the scientific journals lies in its distinctive features. Due to its integrating role at the intersection of cutting‐edge technologies in the field of life sciences and pharmaceutical and healthcare, 2024 is a year full of challenges and opportunities for AMEM. AMEM will continue to play a leading role in the development of laboratory animal science, focusing its efforts on its advantageous and distinctive areas. We will further develop and strengthen the existing themes on brain science, stem cells, multi‐omics data analysis of experimental animals, cardiovascular and cerebrovascular diseases, while at the same time welcoming unconventional contributions reporting original innovations, such as targeted drug prediction, brain‐computer interfaces, and big data analysis. We seek to use these breakthroughs in medical innovation technologies as catalysts for leapfrogging the development of the journal. Developing AMEM themes with distinctive features is an ongoing effort and AMEM is committed to enriching the content of these themes by increasing the number of reviews, short communications and commentaries. As the editor‐in‐chief of AMEM, I encourage you to contribute to the themed sections of the journal and to provide suggestions to improve the development of the sections. With the added benefit of our efficient publishing services, we aim to make AMEM not only a platform for academic exchanges among scientists globally, but also an attractive arena for scientists to showcase their talents, inspirations and blue‐sky thinking.

At the beginning of the Lunar New Year, AMEM once again sincerely invites experts and colleagues from around the world to contribute to AMEM, to make it a hub for academic exchange and discussion. We also extend our thanks to the dedicated efforts of our editorial team throughout the past year and look forward to further collaboration to create outstanding achievements. As we start the new year, the AMEM editorial department will maintain a commitment to excellence and provide high‐quality services to all authors and readers. At the same time, we also welcome and value feedback and suggestions from experts and colleagues globally to jointly promote the development of AMEM. Finally, we wish everyone a happy New Year and good health!

